# Habitat Characteristics as Potential Drivers of the *Angiostrongylus daskalovi* Infection in European Badger (*Meles meles*) Populations

**DOI:** 10.3390/pathogens10060715

**Published:** 2021-06-07

**Authors:** Eszter Nagy, Ildikó Benedek, Attila Zsolnai, Tibor Halász, Ágnes Csivincsik, Virág Ács, Gábor Nagy, Tamás Tari

**Affiliations:** 1 Institute of Wildlife Management and Wildlife Biology, Faculty of Forestry, University of Sopron, H-9400 Sopron, Hungary; nagy.gesztenye07@gmail.com (E.N.); tari.tamas@uni-sopron.hu (T.T.); 2 Institute of Animal Breeding, Kaposvár Campus, Hungarian University of Agriculture and Life Sciences, H-7400 Kaposvár, Hungary; ildiko.benedek@uni-mate.hu (I.B.); attila.zsolnai@gmail.com (A.Z.); 3 Institute of Physiology and Animal Nutrition, Kaposvár Campus, Hungarian University of Agriculture and Life Sciences, H-7400 Kaposvár, Hungary; halasz.tibor@sefag.hu (T.H.); csivincsik.agnes@uni-mate.hu (Á.C.); acs.virag@uni-mate.hu (V.Á.); 4 Somogy County Forest Management and Wood Industry Share Co., H-7400 Kaposvár, Hungary; 5 One Health Working Group, Kaposvár Campus, Hungarian University of Agriculture and Life Sciences, H-7400 Kaposvár, Hungary

**Keywords:** European badger, *Meles meles*, *Angiostrongylus daskalovi*, Hungary

## Abstract

From 2016 to 2020, an investigation was carried out to identify the rate of *Angiostrongylus* spp. infections in European badgers in Hungary. During the study, the hearts and lungs of 50 animals were dissected in order to collect adult worms, the morphometrical characteristics of which were used for species identification. PCR amplification and an 18S rDNA-sequencing analysis were also carried out. Global and local spatial autocorrelation methods were used to detect high-rated and low-rated infected animal clusters. We conducted a binary logistic regression analysis along with hierarchical agglomerative clustering to determine the relation between selected biotic and abiotic variables, and the prevalence of an *A. daskalovi* infection. We found a high prevalence (72%) and moderate mean intensity (14.1) of *Angiostrongylus* sp. infection. Morphology and sequencing revealed that all animals were infected by *A. daskalovi*. The results of both spatial autocorrelations suggested that the spatial distribution of infected badgers was more spatially clustered than random. The results of an analysis of the correlation between habitat characteristics and infection showed that the infected animals could be associated with dry and open landscape habitats without extended and connected canopy. It is suggested that the territorial behaviour of badgers and the landscape-directed aggregation of potential intermediate hosts might be the drivers of an *A. daskalovi* infection.

## 1. Introduction

Members of *the Angiostrongylus* genus, which belongs to the Metastrongyloidea superfamily, often referred to as “lungworms”, may cause severe symptoms in the infected hosts [1]. During their life cycle, they can be found in different definitive hosts, e.g., carnivores, rodents or tupaniids, and in intermediate hosts such as slugs and aquatic or terrestrial snails. In some cases, paratenic hosts may also be involved. First-stage larvae are excreted via faeces and infect the gastropods by ingestion or penetration. In intermediate hosts, the larvae develop into third-stage or infective larvae. Definitive hosts become infected by ingestion of gastropods or their slime [2]. To our present knowledge, out of 22 *Angiostrongylus* species, 6 have been described as infective for carnivores. Of these 22 species, *Angiostrongylus felineus, Angiostrongylus gubernaculatus* and *Angiostrongylus raillieti,* are known in the Americas. *Angiostrongylus chabaudi* and *Angiostrongylus daskalovi* are widespread exclusively on the European continent, while *Angiostrongylus vasorum* also has been isolated in the Americas, Australia and certain parts of Africa. In mustelid species, both *A. vasorum* and *A daskalovi* occur [3,4,5,6].

*A. daskalovi* is a cardiopulmonary parasite of the carnivore taxa, Mustelidae family [4]. The very first report of this nematode dates back to the late 1980s in Bulgaria, where the worm was collected from European badgers (*Meles meles*), European pine martens (*Martes martes*) and beech martes (*Martes foina*) [7]. Since that time, the presence of the *A. daskalovi* has been confirmed in two other countries, Spain, and Romania [8,9]. The parasite populates the lung arteries and the heart’s right ventricle [8]. In previous studies, the prevalence of the *A. daskalovi* infection ranged from 16.95–37.5%, while little is known about the mean intensity (Table 1).

In Hungary from 2009 to 2019, the average number of badgers shot or found dead was 9199 annually. The increase in this number in the past 3 years—10,347 in 2018, 12,394 in 2019, and 16,635 in 2020—indicates the increase of the badger population (National Game Management Database, http://www.ova.info.hu/vgstat.html (accessed on 21 February 2021). Despite this rapid rise of population, only one study with a small sample size was conducted to investigate the parasitological state of the badger population. However, neither the presence of *A. daskalovi* nor that of *A. vasorum* was confirmed [11]. Considering the increase of the definitive host population in Hungary and based on the fact that few adequate data are available on the European situation of the *A. daskalovi*, we carried out a monitoring of the densest mustelid population in southwest Hungary. Our main aim was to determine the biotic and abiotic factors, which may drive angiostrongylosis characteristics in badgers. Our further goal was to estimate the epidemiological risk of an expanding badger population on protected mustelids and companion animals.

## 2. Results

### 2.1. Species Identification

**Figure 1:**
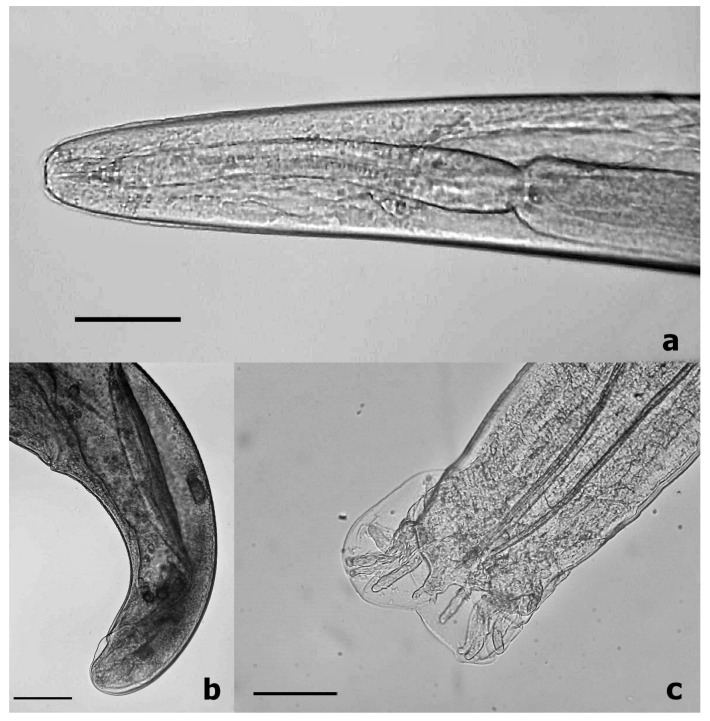
Morphological characteristics of *A. daskalovi* from a badger. The anterior part of the body (**a**), posterior part of a female (**b**), and the bursa copulatrix of a male (**c**), bar: 100 µm.

Among 50 badgers, we found 36 individuals with an *Angiostrongylus* sp. infection; thus, the calculated prevalence was 72% (CI95% = 58.2–83.3%). The worm burden ranged from 1 to 97 specimens (mean intensity: 14.1; CI95% = 10.3–23.2). Based on the observed morphological features, we revealed that all of the animals had an *A. daskalovi* infection and *A. vasorum* was not detected in any specimens (Figure 1). In addition to *A. daskalovi*, three further lungworm taxa (*Crenosoma* spp., *Perostrongylus falciformis*, *Eucoeleus aerophilus*) were observed in the specimens (see Table S1).

**Figure 2:**
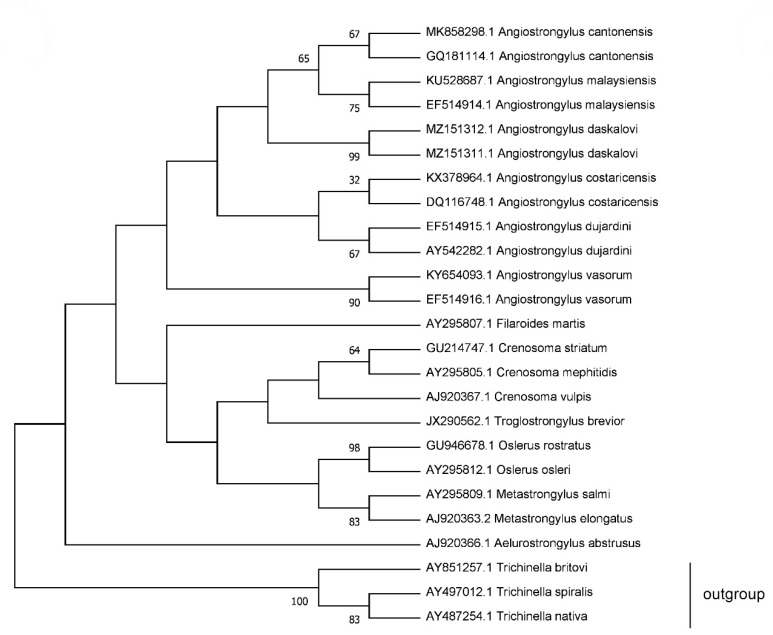
Relationship of *A. daskalovi* with members of the superfamily Metastrongyloidea found by18S rDNA sequencing. (The percentage of replicate trees, in which the associated taxa clustered together in the bootstrap test (50% majority rule consensus with 1000 replicates) is shown next to the branches).

The sequences (GenBank accession no. MZ151311, MZ151312) obtained from two adult *A. daskalovi* males showed 99% of sequence identity. A phylogenetic analysis displayed *A. daskalovi* specimens blending into the clade, including several *Angiostrongylus* spp. sequences available in the GenBank database (Figure 2).

**Figure 3:**
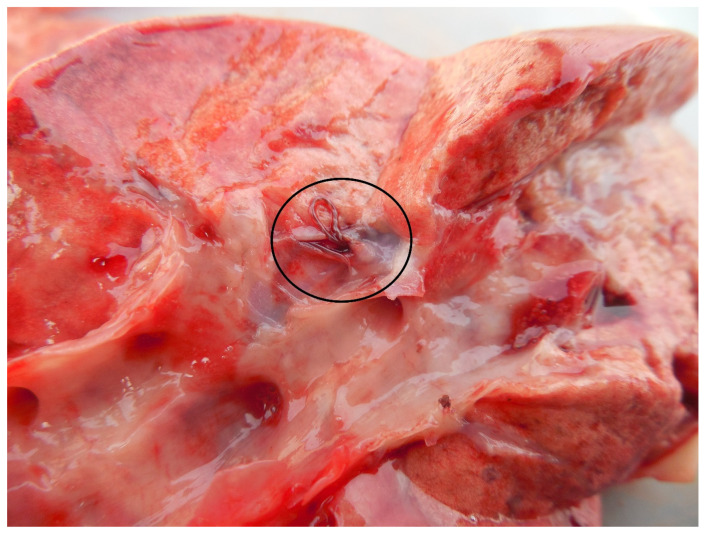
*A. daskalovi* specimen in an opened lung vessel.

Of the infected 36 lung specimens, none showed macroscopically visible lesions of inflammation or other pathological changes (Figure 3).

### 2.2. Spatial Analysis

**Figure 4:**
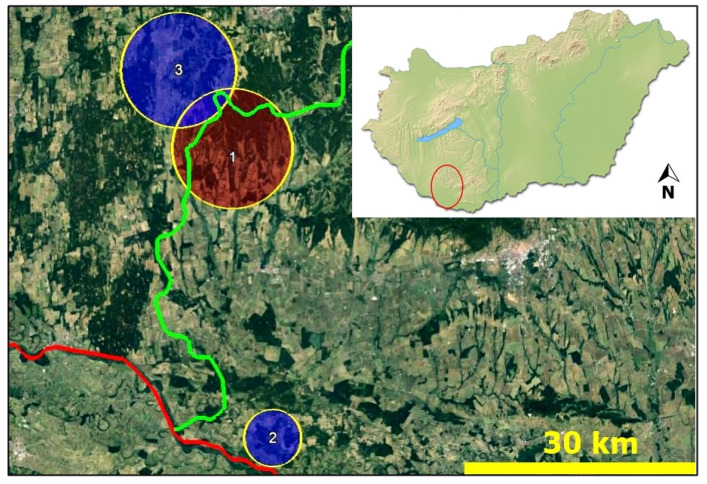
Local spatial clusters of the *A. daskalovi* infection (red = high-rated, significant cluster; blue = low-rated, non-significant cluster; green line = county border; red line = country border).

We collected samples from 24 Universal Transverse Mercator (UTM) quadrates, of which 18 had infected badgers. The Moran’s I (Moran’s I = 0.061; z = 3.0304; *p* = 0.0024) indicated a positive global spatial autocorrelation for the *A. daskalovi*. The result suggested that the spatial distribution of the parasite infection was more spatially clustered than randomly. The Local Spatial Clustering (LSC) disclosed three local clusters (Figure 4). A high-rated cluster proved significant, whereas the two others did not show significance. The radius of the high-rated cluster (*p* = 0.009) was 7.85 km around the centre coordinate (46.188324 N, 17.737759 E). Out of 24 involved badgers, 23 animals were infected; thus, the relative risk proved 1.95 with a 7.47 log-likelihood ratio.

### 2.3. Environmental Determinants of the A. daskalovi Infection

For the *A. daskalovi* infection, nine variables were potential candidates for modelling infection/non-infection. The broad-leaved forest (BLF), precipitation (PREC) and temperature at 2 m in °C (T2M) variables were rejected from the process because their variance inflation factors (VIF) value exceeded the tolerance threshold. The overall model was statistically significant compared to the null model (χ^2^ = 12.416, *p* = 0.006), and justified 31.18% of the variation of infection. It also correctly predicted 78.9% of cases. Our best regression model had a 0.79 area under the curve (AUC) score (*p* = 0.0013) and contained 3 explanatory variables, namely relative humidity at 2 m in % (RH2M), mixed agricultural and forest area (MIX) and wetlands (WET) (Akaike’s Information Criterion, AIC value 56.67). Their regression coefficients (B) suggested a negative relationship between these explanatory variables and the presence of the infection (Table 2).

**Figure 5:**
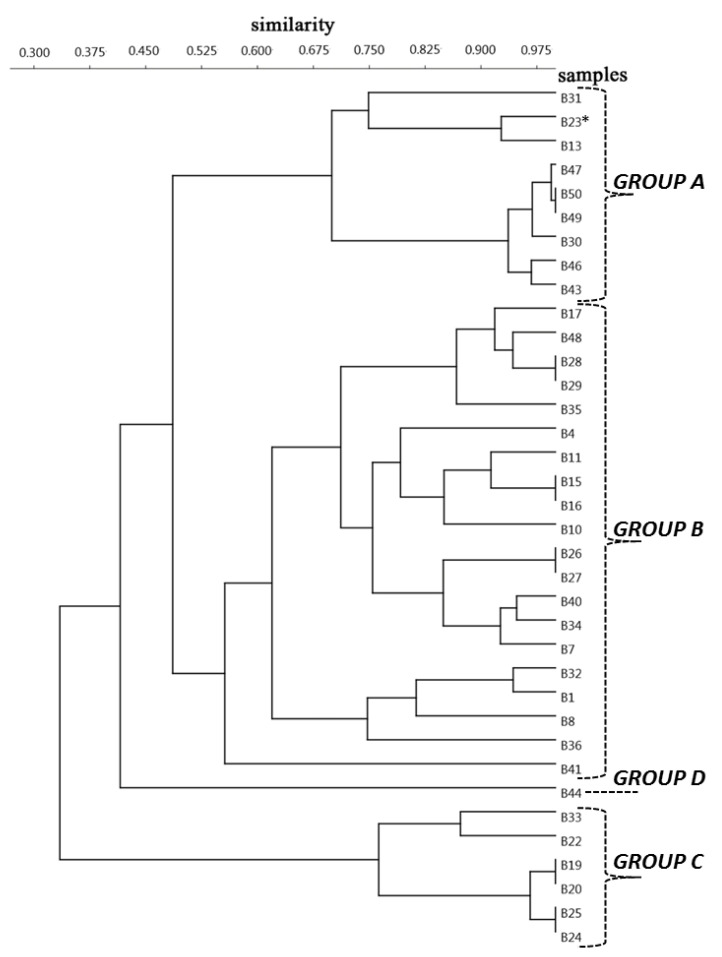
The result of a hierarchical agglomerative clustering analysis of the buffer zone of 36 infected badgers. (*B23 and other identification numbers indicate the infected animals).

By the hierarchical agglomerative clustering (HAC), the 36 infected badgers were divided into 3 main groups and a singleton sample (Figure 5).

**Figure 6:**
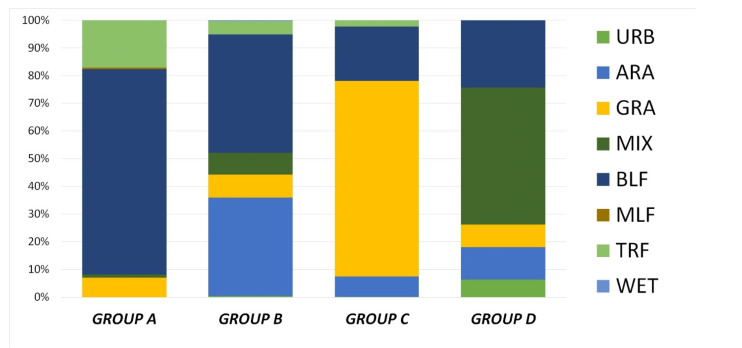
Differences of habitat types among groups were formed by hierarchical agglomerative clustering. (URB = urban area, ARA = arable land, GRA = grassland, MIX = mixed agricultural and forest area, BLF = broad-leaved forest, MLF = mixed forest, TRF = shrubland, WET = wetlands).

Based on similarity values of habitat types in the buffer zones, we determined the predominant habitat categories of different groups. The results showed that the highest proportions of BLF (74.14%) were found in GROUP A. GROUP B was characterised as an agro–forest mosaic habitat with 42.75% BLF and 35.54% arable land (ARA). Grasslands (GRA, 70.65%) dominated in GROUP C, while in GROUP D, the MIX (49.45%) habitat type ruled (Figure 6). Because the last-mentioned group contained only one badger, we did not involve it in further analyses. The landcover difference of habitat types between the groups was confirmed statistically significant (H = 10.95, *p* = 0.004).

In the forest-dominated GROUP A (*n* = 9), the intensity was 9.56 worms/badger. In the agro–forest mosaic structured GROUP B (*n* = 20), the average *A. daskalovi* number was 10.55 worms/badger. In the grassland-dominated GROUP C (*n* = 6), the average parasite number was 34.50 in the infected hosts. The Mann–Whitney pairwise comparison confirmed that the difference between GROUP A and GROUP B was not significant (*p* = 0.0619), while the habitat structure of GROUP C diverged from both GROUP A considerably (*p* = 0.008) and GROUP B (*p* = 0.002).

## 3. Discussion

Our study verified the presence of the *A. daskalovi* in badgers as a new nematode species in Hungary. We observed a surpassingly high prevalence and a moderate mean intensity in the southwestern Hungarian badger population. These results suggested a larger infection rate than observed in previous European surveys [7,8,9,10,11].

Our results may be explained by the dynamic increase of the host population. The larger host groups can result in more intense parasite infections [12]. Growing badger density increases the number of first-stage larvae in the environment, thus in the gastropod intermediate hosts. Finally, this process can promote an elevated parasite prevalence in the host population.

We supposed that the difference in the observed mean intensity is associated with the habitat types, which could influence the feeding habits of badgers. In grassland-dominated habitats, the relevant direct or indirect (with plant parts) consumption of slugs and snails may result in a significant divergence. Consumption of terrestrial gastropods was reported by several authors [13,14,15,16,17,18,19,20,21]. The percentage of this food item in the diet ranges from 0–27.2% and can be influenced by the type of habitat and the season (Table 3). It has to be mentioned that diet analysis studies are frequently based on scat analysis. This noninvasive method may present an error because dietary items composed of soft and highly digestible tissues (e.g., slugs) are not detected properly; thus, an underestimation may occur [22].

Although we did not gather information about the intermediate hosts of the *A. daskalovi* during this study, it is presumable that this species, as the other *Angiostrongylus* spp., involves gastropods in its life cycle as intermediate hosts [4]. Our model showed that as RH2M increases the chances for an infection decrease, while the mean intensity is connected to the proportion of grassland. Both results suggest that badger infection could be connected to terrestrial snails and slugs, which may be found in dry and open landscape habitats without extended and connected canopy. Previous malacological studies revealed the presence of 160 terrestrial gastropod species in Hungary, and more than 70 species could be found in the southwestern region [23]. Several species are strongly associated with grasslands with a moderate or very dense population [24].

We have ample evidence that some of these slugs and snails may be intermediate hosts of *A. vasorum*, *A. andersoni*, *A. dujardini*, and *A. cantonensis* [4,25,26,27]. To our present knowledge, *A. vasorum* larvae may become infectious in *Deroceras agreste* and *Arion lusitanicus* [28,29]. These slugs prefer the mesic open grasslands where the green, plant-based, trophic chain dominates. Due to the adaptive phenotypic plasticity, the invasive *A. lusitanicus* became a dominant species in the previous decade in this region [30]. Its fitness traits and tolerance for heat and cold may help the species to form a very dense population and thus may play a role in the life cycle of *A. daskalovi*. As for snail species, *Helix pomatia* can act as an appropriate intermediate host for the *A. vasorum*. Other species such as *Vallonia pulchella*, *V. costata* and *Trichia hispida* (formerly *Trochulus hispidus*) were described in the life cycle of other metastrongylids [25,29,31,32]; thus, these species may play a role in the biology of *A. daskalovi*.

However, we investigated neither the territorial behaviour of badgers nor the aggregation of potential intermediate hosts. We suggest both might be a driver of an *A. daskalovi* infection. Scent-marking is an evident element in the behaviour of badgers. They use olfactory signals to demarcate their territory and, in their hinterland, sign conspicuous landmarks, such as woodland edges, field margins and fences, etc. by shared defecation sites, the latrines [33,34,35]. Most of these latrines are located very close to vegetation boundaries with linear shape, which characterise grassland-dominated, mosaic-structured landscapes [36,37,38].

In the case of capable intermediate hosts, the natural landmarks may have a similar agglomerative role. A Swiss study revealed that diverse herbal field margins compared to crop margins increased the density and activity of *A. lusitanicus* and *Deroceras* spp. [39]. The authors concluded that these types of margins with their width, type and structure of vegetation cover (e.g., they contain numerous plant species and dead plant material) may contribute to an intensive presence of slugs. We suggest that the higher the proportion of overlap between the territory boundary and field margin is, the higher the prevalence and mean intensity of the parasite might be in both hosts.

## 4. Conclusions

We concluded that the morphological and molecular approach we used in this study definitely identified the *A. daskalovi* infection in Hungarian badger populations. According to the macroscopic pathological findings, this parasite showed apparently moderate impact on individual health status; thus, the determination of population-level effect needs an increased sample size. Our study may contribute to the recognition of some driving factors in the dynamics of badger infections. We deem that the results highlight possible future directions to investigate the finer linkage between definitive and possible intermediate hosts. Based on these findings, we can conclude that further investigation of intermediate hosts in different habitats is needed to discover the factors that influence the life cycle of *A. daskalovi* and the risk it causes for species other than badgers. The sample size of this study was not appropriate to exclude the presence of *A. vasorum* in the concerned badger population: to determine the potential epidemiological role of *A. vasorum*, more specimens should be investigated in the future.

By our molecular analysis (18s rDNA), it seems in the *Angiostrongylus* clade that the *A. vasorum* and *A. daskalovi* form two separated groups. These findings partly differed from others’ work [9,40]. We deem further investigations can clarify the unsettled questions regarding the phylogenetic relationships of *A. daskalovi*.

## 5. Material and Methods

### 5.1. Sample Collection

**Figure 7:**
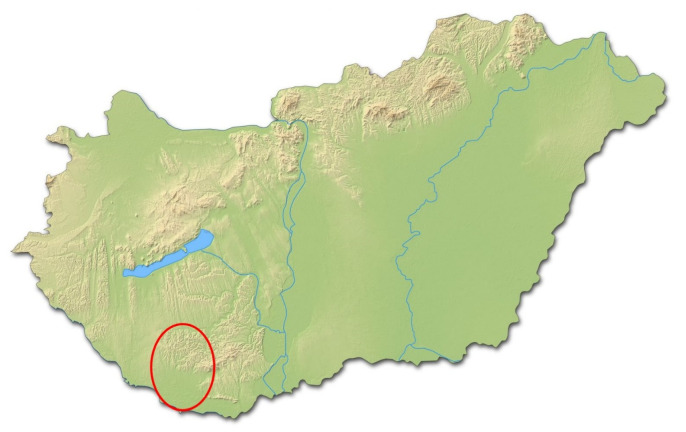
The studied area.

From January 2016 to December 2020 in southwest Hungary, 50 badgers were collected to determine the presence of *A. daskalovi* (Figure 7).

The badgers were either legally hunted with the approval of the national control program for wildlife or were found dead due to vehicle collisions. The animals were necropsied within 24 h after being collected. The lung vessels and the hearts were opened and washed in 0.9% saline solution. The visible pathological lesions, especially focal inflammation, haemorrhage and fibrosis, were evaluated. The collected worms were stored in pure alcohol until their analysis.

### 5.2. Parasitological and Molecular Analysis

At first, we used a morphological approach for species identification. This process was based on the works of Janchev and Genov [7], Gerrikagoitia et al. [8], Gherman et al. [9] and Panayotova-Pencheva et al. [10]. The prevalence and mean intensity were determined with a 95% confidence interval (CI 95%). This analysis was carried out by using the QP web online software version 1.0.15 (http://www2.univet.hu/qpweb/qp10/index.php, accessed on 11 February 2021) [41].

According to the manufacturer’s protocol, genetic DNA samples were extracted using the Qiagen QIAamp DNA Mini Kit (Qiagen, Germany). Our molecular analysis was based on the nuclear 18S rDNA sequences (461 bp). For amplification, we used the primers SSU F07: 5′-AAAGATTAAGCCATGCATG-3′ and SSUR09: 5′-AGCTGGAATTACCGCGGCTG-3′ [41]. The final volume of the reaction mix was 50 µL and consisted of 25 µL PCRBIO Taq Mix (PCR Biosystems, London, UK), 22 µL distilled water, 1 µL of each primer (10 µM) and 1 µL of sample DNA. The PCR cycling conditions were: denaturation at 94 °C for 2 min, 38 cycles of denaturation at 94 °C (for 30 s), annealing at 45 °C (for 30 s), and extension at 65 °C (for 60 s), and a final extension cycles at 72 °C for 4 min. The PCR products were checked by electrophoresis on 1.5% agarose gel. The product purification was carried out by Wizard SV Gel and PCR Clean-Up System (Promega, Madison, WI, USA). The sequences were analysed using ABI 3500 sequencer (Thermo Fisher Scientific, Waltham, MA, USA).

We applied 18S rDNA sequences to determine the phylogenetic relationship between *Angiostrongylus* spp. and other metastrongyloid taxa and to differentiate various species of *Angiostrongylus* genus. The phylogenetic analyses were conducted in MEGA X using the maximum likelihood method and Tamura 3-parameter model with 50% majority rule consensus and 1000 bootstrap replications. The initial tree for the heuristic search was obtained by applying the BioNJ algorithm [42,43].

### 5.3. Spatial Analysis

Spatial autocorrelation (global and local) methods were used to detect infected and non-infected animal clusters. The global clustering analysis of the *A. daskalovi* was evaluated by Moran’s I, and a value was calculated by using 999 permutations. In this case, the null hypothesis (H_0_) stated that the infection was randomly distributed. For Moran’s I determination, we used the 1.18 version of GeoDA software (https://geodacenter.github.io, accessed on 9 March 2021).

To assess the local spatial autocorrelation, we recorded the coordinates where the animals were found dead or shot. Local spatial clustering (LSC) of positive and negative animals was then tested by SaTScan software (version 9.6.1, www.satscan.orgvers accessed on 9 March 2021). We inserted all locations into the 2.5 × 2.5 km Universal Transverse Mercator (UTM) system [44,45]. The presence of the *A. daskalovi* in a host meant a ‘case’, while the non-infected animals formed the ‘control’ group. The analysis also included the centre coordinate of the concerned UTM grids (Table S2). For identifying the significant spatial clusters, we used the Bernoulli Model (purely spatial). The test of significance of the identified clusters is based on a likelihood ratio test and calculated for 999 Monte Carlo simulations with the maximum cluster size of 50% of the total population for parasites [46].

### 5.4. Statistical Analysis

A binary logistic regression analysis was conducted with backward stepwise selection to determine the relation between selected biotic and abiotic variables and the prevalence of the *A. daskalovi* infection. We drew buffer zones around the sampling points of the infected animals (*N* = 36) with a 1.1 km radius [47,48,49,50]. The landcover types within the buffer zones were classified by the CORINE2018 database. We formed seven main habitat types such as urban area (URB, CLC:112), arable land (ARA, CLC: 211), grassland (GRA, CLC: 231), mixed area with similar proportion of agricultural and forest covering (MIX, CLC: 242 and 243), broad-leaved forest (BLF, CLC: 311), mixed forest (MLF, CLC: 313), shrubland (TRF, CLC: 324), and wetlands (WET, CLC: 411 and 512), and determined their proportions in each buffer zone. Climate data, namely relative humidity at 2 m in % (RH2M), precipitation in mm/day (PREC) and temperature at 2 m in °C (T2M), were collected using the sampling coordinates. For the GIS analyses, we used QGIS software [51], while the climatic data were downloaded from the POWER website (https://power.larc.nasa.gov/data-access-viewer/, accessed on 8 March 2021).

The explanatory variables were chosen by using the likelihood ratio test. Before the analysis, we checked the multicollinearity of the variables to eliminate highly correlated explanatory variables. We rejected the variables with the highest variance inflation factor (VIF), if the value exceeded ten [52]. Odds ratios (ORs) and their CI 95% were used to assess the presence and strength of correspondence between the dependent and independent variables. Akaike’s Information Criterion (AIC) was used for assessing different models; lower values indicated a better fitness for the data. The best model performance was assessed by using the area under the curve (AUC). We took the AUC score as a fair one if it was above 0.7. A value above 0.9 indicated that model performance is excellent [53]. The statistical analysis was conducted by SPSS version 22 [54].

During the analysis of the connection between habitat structure and mean intensity, we used the hierarchical agglomerative clustering (HAC) approach to classify all the buffer zones by their characteristics [55]. The categorisation was performed according to the Bray–Curtis dissimilarity index in the PAST3 program [56,57]. The mean intensity of the groups separated by the habitat type was compared by the Kruskal–Wallis test, and differences between groups were analysed using the Mann–Whitney pairwise method. Statistical analyses were performed using the PAST3 program [57].

## Figures and Tables

**Table 1:** Characteristics of *A. daskalovi* Infection in Europe.

Badgers Examined	Badgers Infected	Prevalence (%)	Mean Intensity	Reference
59	10	16.95	4.7	[7]
50	12	24	NA	[8]
8	3	37.5	NA	[9]
11	2	18.18	NA	[10]

**Table 2:** Binary logistic regression for the *A. daskalovi* infection in badgers.

Predictor	B	SD	*p*-Value	OR	OR CI95%	
					Lower	Upper
RH2M	−2.704	±1.112	0.015	0.067	0.008	0.592
MIX	−0.016	±0.008	0.04	0.984	0.968	0.999
WET	−0.093	±0.049	0.056	0.911	0.828	1.002
CONSTANT	199.01	±81.29	0.014	2.69 × 10^86^		

(B = regression coefficient, SD = standard deviation, OR = odds ratio, OR CI95% = 95% confidential interval of odds ratio, RH2M = relative humidity at 2 m, MIX = mixed agricultural and forest area, WET = wetlands).

**Table 3:** The relation between habitat type and proportion of gastropods in badger diet.

Country	Dominating Habitat	Percentage of Occurance	Reference
Hungary (Komárom-Esztergom County)	forest	10.64 *	[13]
	farmland and pastures	1.96 *	
	forest-pasture mosaic	12.0 *	
Italy (Burano Lake Nature Reserve)	Mediterranean maquia	3.85–18.75 **	[14]
Italy (Lombard Prealps)	forest	6.2–11.1 **	[15]
Italy (Piedmont region)	farmland and pastures	0.0–11.4 **	[16]
Poland (Bialowieza National Park)	forest	5.6–25.0 **	[17]
Republic of Ireland	farmland and pastures	1.0–8.0 **	[18]
Spain (southern Iberian Peninsula)	Mediterranean maquia	0.0–27.2 **	[19]
	xeric shrubland	0.0–6.6 **	
	forest	0.0 **	
Spain (northern Iberian Peninsula)	forest and meadows	15.8–27.3 **	[20]
Switzerland (Cantone of Berne)	farmland and pastures	20.0 *	[21]

Badger diet composition was estimated by the analysis of stomachs and/or scats during annual (*) or seasonal (**) investigations.

## Data Availability

Data are contained within the article and its Supplementary Materials.

## Supplementary Materials

The following are available online at https://www.mdpi.com/article/10.3390/pathogens10060715/s1, Table S1: Other collected nematodes from the respiratory system of badgers, Table S2: The coordinates and the parasitological status of collected badgers.
